# Jodhaltige und gadoliniumhaltige Kontrastmittel und Nierenkrankheit: Konsensus-Statement der Österreichischen Gesellschaft für Radiologie (OERG) und der Österreichischen Gesellschaft für Nephrologie (ÖGN)

**DOI:** 10.1007/s00508-026-02822-9

**Published:** 2026-10-27

**Authors:** Gertraud Heinz, Martin Windpessl, Alexandra Ökrösi, Christian Krestan, Katharina Lampichler, Gregor Bond, Marcus Säemann, Katharina Hohenstein-Scheibenecker

**Affiliations:** 1 Institute of Diagnostic and Interventional Radiology, University Hospital St. Pölten – NOE LGA, Karl Landsteiner University, Dunant-Platz 1, 3100 St. Pölten, Austria; 2 Abteilung für Innere Medizin IV, Klinikum Wels-Grieskirchen, Wels, Österreich; 3 Institut für diagnostische und interventionelle Radiologie, Wiener Gesundheitsverbund, Klinik Favoriten, Wien, Österreich; 4 Universitätsklinik für Radiologie und Nuklearmedizin, Medizinische Universität Wien, Wien, Österreich; 5 Universitätsklinik für Innere Medizin III, Abteilung für Nephrologie und Dialyse, Medizinische Universität Wien, Wien, Österreich; 6 Medizinische Abteilung mit Nephrologie & Dialyse, Klinik Ottakring, Wien, Österreich; 7 Universitätsklinik für Orthopädie und Unfallchirurgie, Medizinische Universität Wien, Wien, Österreich; 8 Österreichische Gesellschaft für Nephrologie, https://www.nephrologie.at/

**Keywords:** Kontrastmittelrichtlinien, Iodhaltige Kontrastmittel, Gadoliniumhaltige Kontrastmittel, Chronische Nierenkrankheit, Kontrastmittel-assoziierte akute Nierenschadigung, Contrast media guidelines, Iodine-based contrast media, Gadolinium-based contrast media, Chronic kidney disease (CKD), Contrast-associated acute kidney injury (CA-AKI)

## Abstract

**Hintergrund/Ziel:**

Jodhaltige und gadoliniumhaltige Kontrastmittel sind in der radiologischen Diagnostik unverzichtbar, galten jedoch früher bei chronischer Nierenkrankheit (CKD) wegen potenzieller Nebenwirkungen als problematisch. Studiendaten aus den letzten 15 Jahren führten jedoch zur Erkenntnis, dass das nephrotoxische Potenzial von Kontrastmittel überschätzt wurde und eine Neubewertung angezeigt ist. Ziel dieses Konsensuspapiers ist es, auf Basis von wissenschaftlicher Evidenz und internationalen Guidelines die intravaskuläre Anwendung von Kontrastmitteln bei eingeschränkter Nierenfunktion in praktisch anwendbarer Form zu vereinheitlichen, um zu einer optimalen Diagnosefindung bei maximaler Patientensicherheit zu kommen.

**Methoden:**

Die Österreichische Röntgengesellschaft (OERG) und die Österreichische Gesellschaft für Nephrologie (ÖGN) nominierten jeweils 4 Expertinnen und Experten in eine Arbeitsgruppe zur Erstellung einer Konsensus-Leitlinie für eine sichere Kontrastmittelanwendung bei eingeschränkter Nierenfunktion. Nach einem initialen Treffen dieser Arbeitsgruppe wurde eine Literaturrecherche in verschiedenen medizinischen Datenbanken (PubMed, Embase, Cochrane Central Register of Controlled Trials) durchgeführt, und entsprechende Guidelines verschiedener internationaler Fachgesellschaften wurden identifiziert. Die wissenschaftlichen Arbeiten und Guidelines wurden in 5 weiteren Arbeitsgruppentreffen evaluiert. Die Empfehlungen für die sichere Kontrastmittelanwendung bei CKD wurden im Konsens erarbeitet.

**Ergebnisse/Konsens:**

Die Empfehlungen konnten in 11 Konsensus-Statements zusammengefasst werden. Zusätzlich wurde ein Algorithmus zur Verabreichung von jodhaltigen Kontrastmitteln bei eingeschränkter Nierenfunktion erarbeitet.

**Schlussfolgerung:**

Diese von der OERG und ÖGN erarbeiteten Empfehlungen dienen der Vereinheitlichung intravaskulärer Kontrastmittelverabreichung bei CKD unter Berücksichtigung des diagnostischen Nutzens der kontrastmittelgestützten Untersuchung im Vergleich zum potenziellen Risiko einer damit assoziierten Nierenschädigung.

**Zusatzmaterial Online:**

Eine für den Druck in A4 optimierte Version von Abbildung 1 findet sich im Online Zusatzmaterial. https://doi.org/10.1007/s00508-026-02822-9

## Abstract (en)

**Background/aim:**

Iodinated and gadolinium-based contrast agents are indispensable in radiological diagnostics; however, they were previously considered problematic in patients with chronic kidney disease (CKD) due to potential side effects. Study data from the past 15 years have led to the realization that the nephrotoxic potential of contrast agents has been overstated and that a reassessment is warranted. This consensus paper aims to standardize the intravascular use of contrast agents in the CKD population in a practical manner, based on scientific evidence and international guidelines, enabling both optimal diagnostic yield and maximum patient safety.

**Methods:**

The Austrian Radiological Society (OERG) and the Austrian Society of Nephrology each nominated four experts in a working group to draft consensus guidelines regarding the safe use of contrast agents in patients with impaired kidney function. Following an initial meeting of this working group a literature search was conducted in various medical databases (PubMed, Embase, Cochrane Central Register of Controlled Trials) and pertinent guidelines of various international specialist societies were identified. The scientific work and guidelines were evaluated in five further meetings of the working group. The recommendations for the safe use of contrast agents were compiled in a consensus.

**Results/consensus:**

The recommendations could be summarized in 11 consensus statements. In addition, an algorithm for the administration of iodinated contrast agents in patients with impaired kidney function was created.

**Conclusion:**

This consensus paper, endorsed by the OERG and ÖGN provides a standardized approach for the intravascular administration of contrast agents in individuals with CKD. Emphasis is placed on the diagnostic benefits of a contrast agent-assisted investigation in comparison to the potential risk of an associated kidney damage.

## Einleitung

Kontrastmittel (KM) führen zu einer deutlichen Verbesserung der Bildkontraste und sind derzeit in der radiologischen Diagnostik unverzichtbar. In der klinischen Routine werden v. a. jodhaltige („iodinated contrast media“ [ICM]) und gadoliniumhaltige KM („gadolinium-based contrast agents“ [GBCA]) verwendet. Intravaskuläre KM zählen zu den am häufigsten verwendeten Arzneimitteln und gelten grundsätzlich als sicher, wobei ihr Einsatz mit Nebenwirkungen vergesellschaftet sein kann. In Bezug auf die Nieren wurde bei Vorliegen von Risikofaktoren, insbesondere einer hochgradigen Einschränkung der Nierenfunktion, eine Assoziation zwischen ICM-Gabe und transientem Anstieg des Serumkreatinins (SCr) bzw. tatsächlicher Nierenschädigung beschrieben. Manche GBCAs wurden mit einer fibrosierenden Systemerkrankung – der nephrogenen systemischen Fibrose (NSF) – in Verbindung gebracht. In letzter Zeit hat sich jedoch die Erkenntnis durchgesetzt, dass die Häufigkeit und klinische Relevanz einer KM-assoziierten Nierenschädigung überschätzt wurde [1]. Insbesondere gibt es keine Evidenz für eine bleibende Nierenschädigung im Sinne einer *direkt* aus der ICM-Gabe resultierenden Notwendigkeit einer chronischen Nierenersatztherapie (NET) oder einer erhöhten Mortalität [2–4]. Das Auftreten einer NSF wurde seit der ausschließlichen Verwendung von makrozyklischen GBCA nicht mehr beobachtet. Demgegenüber steht die Erkenntnis, dass der Verzicht auf KM-verstärkte Bildgebung schwerwiegende Konsequenzen haben kann, wie eine verzögerte, falsche oder nicht gestellte Diagnose. Dies schlägt sich bereits in aktuellen internationalen Guidelines zur ICM-Gabe nieder, auf die auch in den 2024 publizierten Leitlinien von KDIGO (*Kidney Disease Improving Global Outcomes*) verwiesen wird [2, 5, 6].

Vor diesem Hintergrund wurde von der Österreichischen Röntgengesellschaft (OERG) und der Österreichischen Gesellschaft für Nephrologie (ÖGN) dieses Konsensus-Statement erarbeitet. Dazu wurde eine Arbeitsgruppe mit jeweils 4 Expertinnen und Experten beider Gesellschaften gebildet, die nach einem initialen Treffen eine Literaturrecherche in medizinischen Datenbanken (PubMed, Embase, Cochrane Central Register of Controlled Trials) durchführte und entsprechende Leitlinien internationaler Fachgesellschaften sichtete. Diese Grundlagen wurden in 5 weiteren Arbeitsgruppentreffen evaluiert. Die 11 Empfehlungen wurden im Konsens erarbeitet und von den Vorstandsmitgliedern der beiden Fachgesellschaften freigegeben.

Ziel dieses interdisziplinären Konsensus ist, die Anwendung von ICM und GBCA in Österreich zu vereinheitlichen, um PatientInnen eine optimale radiologische Diagnostik unter Einbeziehung KM-unterstützter Untersuchungen sicher zu ermöglichen.

Zur Veranschaulichung der Problematik sind in Tab. 1 3 auf realen Szenarien basierende Vignetten dargestellt.

**Tab. 1:** Fallvignetten

Fallvignette 1: Ein 50-jähriger Patient mit einer weit fortgeschrittenen, aber nur langsam progredienten Nierenkrankheit (Serumkreatinin um 5,5 mg/dl; eGFR um 10 ml/min/1,73 m^2^) wird präemptiv für eine Nierentransplantation vorbereitet. Zur kardialen Abklärung wird vom Zentrum eine koronare Diagnostik verlangt. Es erfolgt ein Herz-CT, dabei werden 90 ml Kontrastmittel (KM) Iodixanol verabreicht. Der Kreatininwert beträgt vor der Untersuchung 5,9 mg/dl, 9 Tage danach 5,7 mg/dl und 6 Monate später 5,4 mg/dl. Die Untersuchung ergibt einen unauffälligen Befund, und der Patient kann noch vor Dialysebeginn auf die Warteliste für eine Nierentransplantation aufgenommen werden	(Klinische) Interpretation: Eine kontrastmittelgestützte Untersuchung kann auch bei einer eGFR ≤ 30 ml/min/1,73 m^2^ durchgeführt werden, wenn der diagnostische Nutzen hoch ist oder klinische Entscheidungen maßgeblich beeinflusst werden
Fallvignette 2: Bei einem 65-jährigen Patienten wird ein primär metastasiertes Adenokarzinom diagnostiziert, nach mehreren Linien Chemotherapie ist er 2 Jahre später in kompletter Remission. Bereits zum Diagnosezeitpunkt liegt eine fortgeschrittene Niereninsuffizienz vermutlich multifaktorieller Genese vor (eGFR um 40 ml/min/1,73 m^2^), nach Beendigung der Chemotherapie beträgt der Kreatininwert 1,8 mg/dl (eGFR 36 ml/min/1,73 m^2^). Im Zuge der Nachsorge erfolgen in halbjährlichen Intervallen KM-basierte CT-Untersuchungen, in Summe werden über einen Zeitraum von 5 Jahren 9 derartige Folgeuntersuchungen durchgeführt, bis zuletzt besteht kein Hinweis für ein Tumorrezidiv. Der aktuelle Kreatininwert beträgt 2,2 mg/dl (eGFR 29 ml/min/1,73 m^2^). Der GFR-Verlust liegt im Rahmen der üblichen Abnahme in diesem Alter	Interpretation: Eine Abnahme der Nierenfunktion ist häufig multifaktoriell und kann nicht automatisch wiederholten KM-Gaben zugeschrieben werden
Fallvignette 3: Bei einer 60-jährigen Patientin (Größe 160 cm, Gewicht 50 kg) besteht 13 Jahre nach Nierentransplantation eine weit fortgeschrittene Transplantatinsuffizienz aufgrund einer chronischen Abstoßung. Bei einer anhaltenden eGFR unter 10 ml/min/1,73 m^2^ wird die Entscheidung zur Nierenersatztherapie mittels Peritonealdialyse getroffen, die Restdiurese ist uneingeschränkt und wird wiederholt mit 2 l gemessen. Der Kreatininwert bleibt stabil um 4 mg/dl. Aufgrund einer vorbekannten, größenprogredienten Raumforderung der Transplantatniere (ca. 4 cm) und dem Wunsch auf neuerliche Listung zur Nierentransplantation erfolgt einige Monate später eine elektive Computertomographie mit KM (Kreatinin am Tag der Untersuchung; 4,4 mg/dl), worauf sich die Restnierenfunktion vorübergehend deutlich verschlechtert (Kreatiningipfel 4 Tage nach KM-Gabe: 7,1 mg/dl) und die Patientin auch für einige Tage oligurisch wird, bevor sich Nierenfunktion und Harnmengen wieder auf die Vorwerte rückbilden. Nachdem die Patientin während dieses Zeitraums euvoläm und normotensiv war und keine andere Medikamentenexposition vorlag, wird ein CA-AKI postuliert. Einige Monate später wird die Transplantatniere entfernt, die histologische Aufarbeitung ergibt den Befund eines papillären Nierenzellkarzinoms. Postoperativ wechselt die Patientin an die Hämodialyse	Interpretation: Ein CA-AKI kann, insbesondere bei HochrisikopatientInnen, auch unter Verwendung moderner Kontrastmittel auftreten, ist jedoch häufig reversibel

Interpretation: Ein CA-AKI kann, insbesondere bei HochrisikopatientInnen, auch unter Verwendung moderner Kontrastmittel auftreten, ist jedoch häufig reversibel

## Begriffsdefinitionen in Bezug auf Nierenkrankheit

Zum besseren Verständnis der Anwendung von ICM und GBCA bei akuter und chronischer Nierenkrankheit werden zunächst allgemeine Begriffe erläutert (Tab. 1). Der Terminus „akute Nierenschädigung“ oder „akute Nierenfunktionseinschränkung“ („acute kidney injury“ [AKI]) ist zwar einheitlich definiert und kategorisiert (Tab. 2), diese Einteilung ist aber im klinischen Alltag nur bedingt brauchbar, weil ihr einerseits funktionelle Biomarker (SCr) statt spezifischer Schädigungsmarker zugrunde liegen, andererseits eine Messung anderer Parameter, wie z. B. der Harnmenge, außerhalb von Intensivstationen nicht praktikabel ist. Diese Definition soll daher in erster Linie eine einheitliche Nomenklatur gewährleisten. Ein „AKI“ ist von zahlreichen Faktoren abhängig und daher kein isolierter Laborbefund, sondern v. a. eine klinische Diagnose.

**Tab. 2:** Stadien der AKI bei Erwachsenen (KDIGO)

Stadium	Serumkreatinin	Harnproduktion
1	SCr ≥ 0,3 mg/dl oder SCr-Anstieg um 1,5–1,9× „baseline“	< 0,5 ml/kg/h über 6–12 h
2	SCr-Anstieg um 2,0–2,9× „baseline“	< 0,5 ml/kg/h über ≥ 12 h
3	SCr ≥ 4,0 mg/dl oder SCr-Anstieg ≥ 3,0× „baseline“ oder Notwendigkeit einer Dialyse	< 0,3 ml/kg/h über ≥ 24 h oder Anurie über ≥ 12 h

Die chronische Nierenkrankheit („chronic kidney disease“ [CKD]) wird anhand verschiedener, entweder Kreatinin- und/oder Cystatin C-basierter Schätzformeln (üblicherweise CKD-EPI 2009, zunehmend auch EKFC) und der daraus resultierenden Bestimmung der errechneten glomerulären Filtrationsrate (eGFR) unter Einbeziehung der Albuminurie klassifiziert [7].

## Begriffsdefinitionen in Bezug auf Nierenschäden und Kontrastmittel

In früheren Arbeiten war von KM-induzierter Nephropathie (CIN), KM-Nephropathie oder KM-induziertem AKI (CI-AKI) die Rede, laut Kontrastmittelsicherheitskomitee (CMSC) der Europäischen Gesellschaft für Urogenitale Radiologie (ESUR) definiert als SCr-Anstieg ≥ 0,3 mg/dl (≥ 26,5 μmol) bzw. ≥ 1,5× vom Ausgangswert innerhalb von 48–72 h nach intravaskulärer Applikation von ICM [8]. In rezenten Studien wurden auch andere Definitionen verwendet, in der PRESERVE-Studie (mit über 5000 PatientInnen) beispielsweise ein SCr-Anstieg um zumindest 25 % oder 0,5 mg/dl vom Ausgangswert [9]. Da diese Definitionen rein laborchemischer Natur sind, gelten dieselben Überlegungen wie zum AKI: Eine nur auf Laborparametern basierende Diagnose greift zu kurz. Grundsätzlich wäre eine Vereinheitlichung der Nomenklatur mit den AKI-Kriterien der KDIGO zweckmäßig und wünschenswert, wie z. B. in der aktuellen Guideline des American College of Radiology (ACR) [10].

In rezenteren Arbeiten wurde der kausale Zusammenhang zwischen ICM und SCr-Anstieg infrage gestellt. Bereits 2008 zeigten Newhouse et al., dass frühere Studien zur Inzidenz der „CIN“ am Fehlen einer adäquaten Kontrollgruppe krankten und es bei hospitalisierten PatientInnen ohne KM-Exposition mit ähnlicher Häufigkeit zu einem Anstieg des SCr kam („contrast nephropathy without contrast“) [11]. Dies ist naheliegend, weil PatientInnen, insbesondere jene mit einer hohen Komorbiditätenlast und v. a. auch im Rahmen klinischer Akutereignisse (hämodynamische Instabilität, Exsikkose, nephrotoxische Medikamente als „competitor strikes“), eine größere Schwankungsbreite ihrer eGFR aufweisen können. Außerdem zeigt das SCr bei CKD auch ohne KM-Exposition im Verlauf eine größere Variabilität [12]. Es ist daher nicht möglich, aus einem zeitlichen Zusammenhang kausale Schlüsse zu ziehen (sog. „Post hoc, ergo propter hoc“-Fehlschluss). Anders gesagt, die Indikation für die Bildgebung kann per se Ursache eines AKI sein.

CI-AKI ist definiert als entsprechender Anstieg des Serumkreatinins *und *Fehlen einer anderen Ursache für das AKI. Der Ausschluss einer alternativen Ursache ist in praxi jedoch oftmals nicht möglich [13]. Folglich wird in der modernen Literatur von KM-*assoziiertem* AKI (CA-AKI) gesprochen. Da der kausale Zusammenhang zwischen Anstieg des SCr – fälschlicherweise in der Literatur immer wieder „Nierenfunktionsverschlechterung“ genannt – und KM-Administration oft nicht eindeutig ist, wurde früher empfohlen, die Begriffe CI-AKI und CA-AKI durch den Begriff „post-contrast AKI“ (PC-AKI) zu ersetzen, wenn es innerhalb von 48 h nach intravaskulärer Gabe von ICM zu einem Anstieg der Retentionsparameter kommt, also die Zeitabfolge im Vordergrund steht [14]. Zuletzt wurde die Nomenklatur in den internationalen Guidelines jedoch zugunsten des Begriffes CA-AKI vereinheitlicht [8, 10]. Jedenfalls sollten Ursachen-unabhängige („cause agnostic“) Begriffe verwendet werden. Eine Übersicht über diese Begriffe bietet Tab. 3.

**Tab. 3:** Definitionen

Begriff	Definition	Kommentar
Akute Nierenfunktionseinschränkung (AKI)	Anstieg des Serumkreatinins ≥ 0,3 mg/dl (≥ 26,5 μmol/l) innerhalb von 48 h. Anstieg des Serumkreatinins auf ≥ 1,5-Fache des Ausgangswerts der letzten 7 Tage Urinvolumen ≤ 0,5 ml/kg/h für mindestens 6 h	Definiert auf Basis der KDIGO-Kriterien, mit Einstufung nach Schweregrad I–III
Chronische Nierenerkrankung (CKD)	Anomalien der Nierenstruktur oder -funktion ≥ 3 Monate lang bestehen mit Auswirkungen auf die Gesundheit	Definiert auf Basis der KDIGO-Kriterien, mit Einstufung nach Ursache, Kategorie der glomerulären Filtrationsrate (eGFR) (G1–G5) und Kategorie der Albuminurie (A1–A3)
Kontrastmittelassoziiertes AKI (CA-AKI)	AKI nach Kontrastmittelgabe, umfasst CI-AKI und andere Ursachen von AKI	Der Begriff „assoziiert“ setzt keine Kausalität voraus, sondern beschreibt lediglich den zeitlichen Zusammenhang zwischen ICM-Gabe und AKI
Postcontrast acute kidney injury (PC-AKI)	AKI nach Kontrastmittelgabe, in der Definition gleich wie CA-AKI	Hier steht die Chronologie und nicht die Kausalität im Vordergrund
Kontrastmittelinduziertes AKI (CI-AKI)	AKI nach Kontrastmittelgabe das auf einen durch Kontrastmittel verursachten Nierenschaden zurückzuführen ist	Die Definition geht davon aus, dass Kontrastmittel AKI verursacht hat, was heute als sehr selten und/oder als unbewiesene Kausalität gilt
Kontrastmittelinduzierte Nephropathie (CIN)	Anstieg des Kreatinins um 44 μmol/l oder 25 % vom Ausgangswert 24–72 h nach Kontrastmittelgabe	In der älteren Literatur zu finden, impliziert eine Kausalität, die unbewiesen bleibt

## Einfluss von jodhaltigen Kontrastmitteln auf die Niere

Ein nephrotoxisches Potenzial von ICM wurde erstmals in der Mitte des 20. Jahrhunderts postuliert [15]. Bis in die 1980er-Jahre wurden ausschließlich hochosmolare Präparate verwendet (ca. 1500 mOsm/kg), die ein genuines renales Toxizitätspotenzial aufwiesen. In der Folge wurden niedrig- („low osmolar contrast material“ [LOCM], 500–850 mOsm/kg) oder isoosmolare Kontrastmittel („iso-osmolar contrast media“ [IOCM], 290 mOsm/kg; einziger verfügbarer Vertreter: Iodixanol) eingeführt, welche heutzutage weltweit zum Einsatz kommen.

Experimentell und theoretisch wurden verschiedene Hypothesen zur Pathophysiologie einer möglichen ICM-Nephrotoxizität vorgeschlagen, insbesondere Änderungen der glomerulären Hämodynamik (Vasokonstriktion) und/oder eine direkte Tubulustoxizität [16]. Letztere wäre durch die deutliche tubuläre Konzentrationszunahme erklärbar, bei stark eingeschränkter Nierenfunktion kann das KM in (nativen) Folgeuntersuchungen noch Tage nach der Applikation nachweisbar sein (*Nephrogramm*) [13, 17]. Allerdings konnte in keinem Modell eine Kausalität zwischen ICM und Nephrotoxizität nachgewiesen werden [18–28]. So lässt sich im Tierversuch eine Nierenschädigung durch ICM nur nach Vorbehandlung der Versuchstiere mittels Dehydrierung und/oder Gabe von hoch dosierten nichtsteroidalen Antirheumatika (NSAR) bewerkstelligen, zudem werden in diesem Modell exzessiv hohe ICM-Dosen benötigt, um renale Effekte zu demonstrieren [29]. Obwohl im klinischen Alltag derartige „Multi-hit-Szenarien“, insbesondere bei hospitalisierten PatientInnen, wohl auch vorkommen können, gilt es hier zuvorderst, die modifizierbaren Faktoren zu behandeln (Absetzen von NSAR, Korrektur einer Exsikkose …).

CA-AKI ist durch eine vorübergehende Abnahme der GFR charakterisiert, wobei die meisten PatientInnen asymptomatisch bleiben und sich die Nierenfunktion meist binnen einer Woche wieder spontan normalisiert. Dies unterstützt die Annahme, dass ein etwaiger SCr-Anstieg nach KM-Gabe in erster Linie auf hämodynamische Effekte zurückzuführen ist [30]. Schließlich fand sich in Biomarkersubstudien von PRESERVE zwischen PatientInnen mit oder ohne CA-AKI kein Unterschied im Verlauf genuiner tubulärer Schädigungsmarker (wie der im Urin nachweisbaren Zellzyklus-Arrest-Proteine TIMP‑2 und IGFBP7 [als NephroCheck® kommerziell verfügbar]) [30, 31].

CA-AKI kann jedoch auch mit ungünstigen klinischen Konsequenzen assoziiert sein, wobei unklar ist, ob dies nicht in erster Linie auf zugrunde liegende, von der KM-Gabe unabhängige, Komorbiditäten zurückzuführen ist [3, 4, 32]. Einzelne Fallserien, die von einer dauerhaften Beeinträchtigung der Nierenfunktion nach KM-Gabe berichten, liegen Jahrzehnte (aus der Ära der ältesten KM-Generation) zurück und weisen erhebliche Limitationen auf [33].

## Risikostratifizierung

Als Risikofaktoren für ein CA-AKI werden eine vorbestehende Nierenkrankheit –insbesondere im CKD-Stadium 4 und 5 (eGFR ≤ 30 ml/min/1,73 m^2^) – und ein koinzidentes AKI angesehen [34, 35]. Die Effektstärke scheint jedoch sehr gering zu sein. Eine retrospektive Arbeit zeigte, dass selbst bei PatientInnen im Stadium CKD 5 (*N* = 76; mediane eGFR 13 ml/min/1,73 m^2^) die intraarterielle KM-Gabe (elektive Koronarangiographie zur Abklärung vor Nierentransplantation) einen eGFR-Verlust nicht beschleunigte; das kumulative dialysefreie Überleben betrug nach 6 Monaten fast 90 % [36]. Ein ähnliches Ergebnis fand sich in einer prospektiven Studie in einem vergleichbaren Kontext [37]. Schließlich kam es in der CARE-Studie (randomisierter Vergleich von LOCM vs. IOCM bei Koronarangiographie, kein Unterschied bezüglich „CIN“-Inzidenz) bei keinem der 414 PatientInnen, von denen 32 eine eGFR < 30 ml/min/1,73 m^2^ aufwiesen und 40 % Diabetiker waren, zu einer dialysepflichtigen Nierenschädigung. Die unveränderte Kategorisierung dieser Population als „high-risk“ in aktuellen Publikationen ist daher zumindest zu hinterfragen [5].

In Studien werden zusätzlich ein höheres Lebensalter, kardiovaskuläre Begleiterkrankungen, Diabetes mellitus (als Verstärker ab CKD 3) und Anämie als Risikofaktoren angeführt [9, 38]. Auch in einer rezenten Metaanalyse werden, wieder bei zugrunde liegender CKD, das Lebensalter und bestehende kardiovaskuläre Vorerkrankungen als wesentliche Prädiktoren für ein CA-AKI genannt [39]. Diese Faktoren sind auch Bestandteile eines adaptierten Risikoscores vor Koronarinterventionen, der von Mehran et al. 2021 publiziert wurde [40].

Neben diesen patientenbezogenen Faktoren beeinflussen auch Art und Umstände der ICM-Gabe das Risiko. Eine intraarterielle (i.a.) ICM-Administration ist mit einem höheren Risiko für ein CA-AKI assoziiert als eine intravenöse (i.v.) Gabe [2, 41]. Interventionelle Untersuchungen weisen ein höheres Risiko auf als rein diagnostische, wobei hier zusätzliche prozedurale Faktoren der Katheterintervention ins Spiel kommen wie durchschnittlich längere Eingriffsdauer (und damit verbunden höhere KM-Volumina). Weiters ist davon auszugehen, dass Akutinterventionen mit einer höheren Rate an Kreislaufinstabilität vergesellschaftet sind. Ein radialer Zugangsweg weist ein geringeres Risko für AKI auf als ein femoraler, was unter anderem auf eine niedrigere Rate an Blutungskomplikationen und Cholesterinembolien zurückgeführt wird [42]. Bei der i.a.-Applikation ist auch zu beachten, ob es sich um eine i.a.-Injektion mit „first pass renal exposure“ oder „second pass renal exposure“ handelt. Im ersten Fall erreicht das ICM die Nierenarterien in (nahezu) unverdünnter Form (z. B. bei Injektion des ICM in das linke Herz, die thorakale bzw. suprarenale Aorta oder direkt in die Nierenarterien). Beim „second pass“ kommt das ICM nach pulmonaler oder peripherer Zirkulation bereits verdünnt in den Nierenarterien an (z. B. bei Injektion im Rechtsherz, in den Pulmonalarterien, den Karotiden, den Koronararterien und kaudal der Nierenarterien) [3].

Grundsätzlich bleiben diese Überlegungen jedoch hypothetisch und es wird für diagnostische Verfahren, die eine i.a.-Gabe von ICM erfordern, ein ähnlicher Ansatz wie bei der i.v.-Applikation empfohlen. Bei therapeutischen Verfahren, die eine i.a.-Applikation von ICM erfordern, sind Alternativen außerdem selten verfügbar oder klinisch angemessen und die Verabreichung von ICM kann selbst in Hochrisikosituationen (hämodynamische Instabilität, schwerwiegende Begleiterkrankungen …) erforderlich sein [9]. Der klinische Kontext (wie z. B. die i.a.-KM-Gabe im Rahmen eines Koronarsyndroms vs. die i.v.-KM-Gabe bei einer ambulanten CT-Untersuchung) ist somit für die Risikostratifizierung von zentraler Bedeutung.

## Dosis und Art des KM

Wiederholte ICM-Gaben und höhere KM-Dosen werden mit einem höheren Risiko für CA-AKI in Verbindung gebracht [10, 43, 44]. Potenzial für Bias ergibt sich daraus, dass insbesondere technisch komplizierte Interventionen in komplexen klinischen Szenarien oftmals höhere KM-Dosen erforderlich machen [3]. Darüber hinaus konnte durch die in den letzten Jahren verbesserte CT-Technologie und den Einsatz entsprechender Software die KM-Menge im Vergleich zu früher reduziert und weiter optimiert werden. Auch vaskuläre Interventionen können nun mit reduzierten KM-Mengen durchgeführt werden, weil üblicherweise die präinterventionelle Diagnostik mittels CT- oder MR-Angiographie erfolgt. Im Gegensatz zu Untersuchungen mit i.a.-KM-Gabe gibt es bei i.v.-KM-Applikationen keine Belege für ein volumenbezogenes renales Risiko, womöglich auch, weil die entsprechenden Volumina heutzutage durchwegs unter 100 ml liegen [5, 10].

Im 2020 publizierten ACR-Konsensus wird bei i.v.-Verabreichung von KM explizit von einer Dosisreduktion abgeraten („contrast media dose reduction … should be avoided“), weil dies Potenzial für suboptimale Ergebnisse birgt [5].

Auch die physikalisch-chemischen Eigenschaften der ICM spielen eine Rolle. Bezüglich der Diskussion, ob IOCM gegenüber LOCM Vorteile bringen, gibt es unterschiedliche Daten in der Literatur [45–47]. Tendenziell geht aber aus der neueren Studienlage hervor, dass kein signifikanter Unterschied bestehen dürfte. Daher kann die Auswahl des entsprechen ICM auf Basis anderer Überlegungen (z. B. Kosten, Verfügbarkeit, Verträglichkeit) erfolgen [2].

## Screening von Risikofaktoren für CA-AK

Das Screening sollte das Erkennen von PatientInnen mit vernachlässigbarem Risiko ermöglichen, ohne Verzögerungen einer medizinisch indizierten intravaskulären Applikation von ICM und ohne übermäßige Kosten zu verursachen, v. a. sollte es praktikabel sein [2, 8]. Gemäß den 2024 aktualisierten Empfehlungen des *National Institute for Health and Care Excellence* (NICE) sollte im ambulanten Rahmen vor einer elektiven Untersuchung mit ICM erfragt werden, ob eine CKD bekannt ist, eine nephrologische Betreuung besteht oder ob eine akute Erkrankung vorliegt, die ein AKI begünstigen könnte (angeführt werden Hypovolämie, Infektionen oder Harnverhalt) [48]. Ist keiner dieser Punkte vorhanden, kann laut NICE im ambulanten Setting die ICM-Untersuchung auch ohne Kenntnis von SCr (bzw. eGFR) durchgeführt werden. Bei Vorliegen von Risikofaktoren ist vor einer elektiven ICM-Administration eine Bestimmung der Nierenwerte zweckmäßig, als Zeitrahmen gibt NICE 6 Monate an, auch der kanadische Konsensus liegt in dieser zeitlichen Dimension (3 bis 6 Monate) [2]. Wenngleich diese Empfehlungen keine wissenschaftliche Grundlage haben, halten wir diese Zugangsweise für pragmatisch, zumal die meisten PatientInnen, die eine KM-basierte CT-Untersuchung im ambulanten Bereich benötigen, a priori ein geringeres Risikoprofil aufweisen. In vielen Fällen werden um den Zeitpunkt der Indikationsstellung für eine Bildgebung auch Laborwerte bestimmt. Das Szenario, in dem ein/e Patient/in zu einer ambulanten Untersuchung mit florider Infektion, exsikkiert oder im akuten Harnverhalt erscheint, hält die Arbeitsgruppe allerdings für konstruiert und schlägt daher vor, das Screening auf die einfache Frage nach CKD und Nierentransplantation zu beschränken, wie auch in den kanadischen Empfehlungen empfohlen wird [2].

**Abb. 1:**
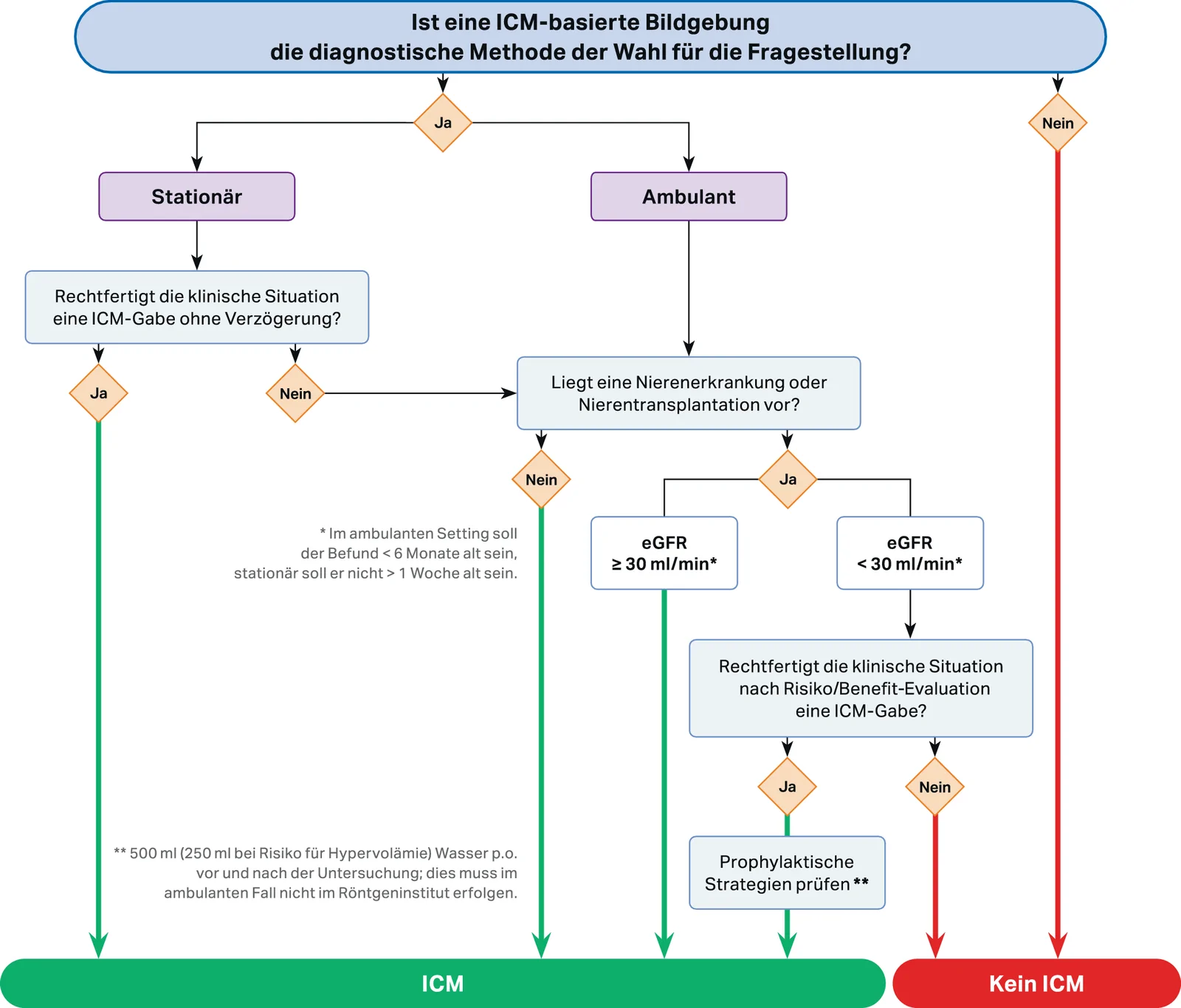
Algorithmus zur ICM-basierten Bildgebung. eGFR, errechnete glomeruläre Filtrationsrate; ICM, Jodhaltige Kontrastmittel; ml, Milliliter; p.o., oral

Im akuten Setting kann nach Nutzen- und Risiko-Abwägung der ICM-Gabe und, wenn möglich bzw. vertretbar, durch eine Besprechung mit den PatientInnen und/oder den Angehörigen, auf eine Bestimmung der Nierenwerte verzichtet werden. Es gilt jedenfalls, dass eine dringliche Untersuchung mit ICM durch die Bestimmung von Laborparametern nicht verzögert werden sollte (Abb. 1; [48]).

## PatientInnen mit akuter Nierenfunktionseinschränkung (AKI)

Bei PatientInnen mit AKI ist eine Risikostratifizierung anhand der Nierenfunktion nur eingeschränkt möglich, weil eine punktuelle eGFR-Messung nicht die aktuelle Nierenfunktion widerspiegelt (die eGFR gilt nur im „steady-state“). Während PatientInnen mit AKI möglicherweise anfälliger für Nephrotoxin-induzierte Nierenschäden sind als PatientInnen ohne AKI, gibt es keine kontrollierten Studien zu diesem Risiko. In einer rezenten retrospektiven Arbeit mit großer Fallzahl unterschied sich das „renale Outcome“ von PatientInnen, die zum Aufnahmezeitpunkt in der Notaufnahme bereits ein AKI hatten und ICM erhielten, nicht von der Kontrollgruppe ohne KM-Exposition [49]. Das theoretische Risiko einer dauerhaften CKD nach ICM-Administration muss bei diesen PatientInnen gegen das Risiko von verspätet erfolgten oder verpassten Diagnosen, die durch die fehlende Verwendung von KM verursacht werden, abgewogen werden. Krankheitsbilder, die im Kontext eines AKI eine KM-basierte Untersuchung notwendig machen, sind zudem meist schwerwiegend, oft existiert keine gleichwertige Methodik, um die klinische Fragestellung zu beantworten [50].

## PatientInnen mit Nierenersatztherapie

Bei HämodialysepatientInnen besteht keine Notwendigkeit, den Dialyseplan wegen einer erfolgten oder geplanten ICM-Applikation zu ändern [2, 8]. Bleibende Auswirkungen auf eine allfällige Restdiurese ließen sich in mehreren Studien nicht nachweisen [51]. Eine systematische Überprüfung von neuen Studien zeigt, dass ICM nur geringe oder nur vorübergehende Auswirkungen auf die verbleibende Restdiurese haben, weshalb eine Restdiurese keine Kontraindikation für eine ICM-Administration in dieser Population darstellt [2, 51, 52].

## Wiederholte Gabe von Kontrastmitteln

In diesem Abschnitt wird aufgrund der Überlappungen sowohl auf ICM als auch GBCA eingegangen, eine ausführliche Behandlung von GBCA erfolgt in einem eigenen Abschnitt.

Bei nierengesunden Personen beträgt die Halbwertzeit (T/2) von gängigen ICM etwa 2 h, bei Abnahme der Nierenfunktion nimmt die T/2 kontinuierlich zu (bis 24 h bei schwerer Niereninsuffizienz) [53]. In spezifischen Situationen kann eine wiederholte Gabe von KM notwendig sein, um eine sichere Diagnose zu erstellen oder eine Behandlung durchzuführen [54].

In einer rezenten Publikation des CMSC der ESUR wird empfohlen, bei elektiven Untersuchungen folgende Wartezeiten zwischen wiederholten ICM-Applikationen zu erwägen („consider“) [53]:

- eGFR > 60 ml/min/1,73 m^2^: 4–12 h,
- eGFR > 30–60 ml/min/1,73 m^2^: 16–48 h,
- eGFR < 30 ml/min/1,73 m^2^: 2,5 bis 7 Tage.

Die gleiche Empfehlung wird auch bei wiederholter Administration von GBCA ausgesprochen [53].

Als empfohlener Abstand bei eGFR > 60 ml/min/1,73 m^2^ zwischen 2 elektiven Untersuchungen mit jeweils ICM oder GBCA werden mehr als 4 h angegeben.

In seltenen Fällen kann die zeitnahe Untersuchung mit einem ICM *und* einem GBCA erforderlich sein. Hier werden von der ESUR folgende Empfehlungen gemacht [53]:

- GBCA vor ICM (Ausnahme: CT-Urographie),
- eGFR > 60 ml/min/1,73 m^2^: 2–6 h,
- eGFR > 30–60 ml/min/1,73 m^2^: 16–48 h,
- eGFR ≤ 30 ml/min/1,73 m^2^: 2,5 bis 7 Tage.

Diese Empfehlungen basieren auf pharmakokinetischen Daten, verwiesen wird auf potenzielle Sicherheitsaspekte („potential safety issues“), Belege für klinisch relevante Endpunkte werden nicht angeführt. Eine strikte Befolgung kann unerwünschte logistische Konsequenzen nach sich ziehen. Beispielsweise sind im Rahmen von (v. a. ambulanten) Tumornachsorgen sequenzielle CT- und MR-Untersuchungen nicht ungewöhnlich, für PatientInnen mit einer CKD im Stadium 3 würde sich aus den ESUR-Vorgaben zwischen den Untersuchungen ein Mindestabstand von 16 h ableiten, was unpraktikabel ist.

## Präventive CA-AKI-Maßnahmen (sog. *Prophylaxe*)

Es existieren zurzeit keine pharmakologischen Substanzen, die zur Vorbeugung einer CA-AKI geeignet sind. Insbesondere wird der prophylaktische Einsatz von N‑Acetylcystein (NAC) vor ICM-Gabe spätestens seit Publikation der PRESERVE-Studie nicht mehr empfohlen [2, 9, 55, 56]. Auch Natriumbikarbonat bietet im Vergleich zu 0,9 %-NaCl-Lösungen keine Vorteile [9].

### Hydrierung

In Stellungnahmen maßgeblicher Gesellschaften wird bei PatientInnen mit einer eGFR ≥ 30 ml/min/1,73 m^2^ keine Hydrierung mehr empfohlen [2, 5]. Konkret zeigten 2 prospektive randomisierte interventionelle Arbeiten (AMACING und KOMPAS; jeweils in einem Non-inferiority-Design), dass in dieser Population das Risiko für CA-AKI vernachlässigbar gering ist, eine Hydrierung aber mit potenziell schwerwiegenden Konsequenzen behaftet ist [38, 57]. So kam es in der monozentrischen AMACING-Studie bei „RisikopatientInnen“ (*N* = 660, Durchschnittsalter 72 Jahre, mediane eGFR 47 ml/min/1,73 m^2^) bei elektiven ICM-basierten Untersuchungen (über 90 % ambulante PatientInnen) im Hydrierungsarm (0,9 %-NaCl-Lösung, durchschnittliches Volumen: 1637 ml) zu 13 Episoden (4 %) von symptomatischer Herzinsuffizienz, während im Kontrollarm (ohne Volumengabe) keine Komplikationen auftraten; in der gesamten Studienkohorte waren in der Nachbeobachtung (etwa ein Monat) keine Fälle von Dialysepflichtigkeit zu verzeichnen. Fast die Hälfte der Untersuchungen erfolgte zudem mit i.a.-ICM [38]. Diese Ergebnisse sind von hoher Relevanz, weil sie verdeutlichen, dass insbesondere bei PatientInnen, die aufgrund von Alter und/oder Komorbiditäten (über 70 % hatten kardiovaskuläre Begleiterkrankungen) als Risikopopulation galten, eine periprozedurale Flüssigkeitsgabe nichts nützt, sondern sogar schaden kann.

Die multizentrische KOMPAS-Studie zeigte in einer Kohorte ähnlichen Alters, die vergleichbare Begleiterkrankungen aufwies (ambulante PatientInnen, ausschließlich intravenöse KM-Gabe), dass die Gabe von 250 ml 1,4 % Natriumbikarbonat keinen Vorteil gegenüber keiner Prähydrierung erbrachte; auch in dieser Studie benötigte kein/e PatientIn während der Nachbeobachtung eine Dialyse [57]. Auch KOMPAS belegt somit die Sicherheit einer konservativen Vorgehensweise für elektive Untersuchungen bei PatientInnen mit einer eGFR ≥ 30 ml/min/1,73 m^2^.

Da bei dringlicher Indikation ohne Alternative eine ICM-Gabe immer erfolgen sollte, verbleiben als klinisch relevante Gruppe, für die die ideale Vorgehensweise offen ist, PatientInnen mit einer eGFR < 30 ml/min/1,73 m^2^ (CKD 4 und 5, Nicht-Dialyse), bei denen eine KM-basierte Untersuchung zwar nicht aus vitaler Indikation indiziert, aber trotzdem von prognostischer Bedeutung ist (z. B. Staging-Untersuchungen bei Personen mit onkologischen Erkrankungen). In dieser Konstellation ist die Datenlage limitiert, und Empfehlungen können daher nur ohne ausreichende Evidenz ausgesprochen werden. Trotzdem ist es Ziel der Arbeitsgruppe, diesen Graubereich im vorliegenden Konsensus zu erfassen, um auch diesen PatientInnen einen möglichst sicheren und zeitgerechten Zugang zu klinisch indizierten Untersuchungen zu ermöglichen. Eine sorgfältige Abwägung von erwartbarem Nutzen und potenziellem Schaden ist selbstredend, Für und Wider einer Untersuchung sollten mit PatientInnen besprochen werden [2].

Obwohl auch für PatientInnen mit einer eGFR < 30 ml/min/1,73 m^2^ keine Belege für den Nutzen einer Flüssigkeitsgabe in Bezug auf renale Endpunkte vorliegen, wird diese Strategie weithin von Guidelines empfohlen [2, 8, 10]. Dabei ist es wichtig zu erwähnen, dass PatientInnen mit fortgeschrittener CKD deutlich mehr kardiale Begleiterkrankungen aufweisen (z. B. Vorhofflimmern, pulmonale Hypertension etc.), insbesondere steigen Prävalenz und Schweregrad einer Herzinsuffizienz mit fortschreitendem CKD-Stadium drastisch an, sodass hier eine Volumenexpansion zu einer noch höheren Rate an kardialer Dekompensation führen könnte und daher erst nach besonders sorgfältiger Prüfung erfolgen darf. Die AutorInnen der AMACING-Studie zeigten demnach auch in einer Folgepublikation, dass bei 18 von 281 PatientInnen mit CKD-Stadium 4 und 5 im Hydrierungsarm Komplikationen auftraten, eine Rate, die noch höher war als bei PatientInnen mit früheren CKD-Stadien. Prädiktoren dafür waren stationär durchgeführte Untersuchungen, Alter > 75 Jahre, kardiovaskuläre Vorerkrankungen sowie Einnahme von Diuretika (vermutlich auch als Surrogat für eine Herzinsuffizienz) [58]. Umgekehrt scheint das Risiko einer Flüssigkeitsgabe bei jüngeren PatientInnen ohne kardiovaskuläre Begleiterkrankungen, die eine ambulante CT-Untersuchung benötigen, intuitiv vertretbar, zumal die entsprechenden Flüssigkeitsmengen nicht wesentlich von einer „normalen“ Trinkmenge abweichen.

Zur Hydrierung kommt entweder eine intravenöse oder eine orale Flüssigkeitszufuhr in Betracht. Eine retrospektive spanische Publikation fand keinen Vorteil einer parenteralen Hydrierung im Vergleich zu einer oralen Flüssigkeitszufuhr bei ambulanten Untersuchungen bei Personen mit CKD 3b [59]. Für PatientInnen mit weiter fortgeschrittener CKD liegen keine Daten vor [10]. Eine bekannte Übersichtsarbeit sieht die orale Hydrierung als zumindest gleich wirksam wie eine i.v.-0,9 %-NaCl-Lösung [16] Auch ein systematischer Review fand keinen Unterschied. In der laufenden dänischen ENRICH-Studie wird genau dieser Frage nachgegangen, indem bei PatientInnen mit einer eGFR < 30 ml/min/1,73 m^2^ vor i.v.-ICM-Gabe entweder eine orale oder parenterale Flüssigkeitsgabe erfolgt.

Anerkennend, dass es für den Nutzen einer Hydrierung bei CKD 4–5 derzeit keine ausreichende Evidenz gibt, jedoch mögliche Komplikationen und im Einklang mit den Empfehlungen der kanadischen Arbeitsgruppe („institutions may choose practices best suited to their local environments“) erachten wir folgendes pragmatisches „Protokoll“ für einen zweckmäßigen Ansatz: Etwa 1 h vor der geplanten Untersuchung werden 500 ml Wasser getrunken, weitere 500 ml unmittelbar nach der Untersuchung; bei bekannter Herzinsuffizienz oder anderen kardiovaskulären Komorbiditäten soll die Flüssigkeitsmenge auf je 250 ml vor bzw. nach der Untersuchung reduziert werden [60]. Entschließt man sich aufgrund des klinischen Eindrucks zu einer Hydrierung, aber eine p.o.-Flüssigkeitszufuhr ist nicht möglich, sollte das periprozedurale Volumen auf 2-mal 500 ml 0,9 %-NaCl-Lösung beschränkt werden (entsprechend dem durchschnittlichen Volumen, das in der PRESERVE-Studie verabreicht wurde) [9].

Zusammenfassend spricht sich die Arbeitsgruppe gegen eine pauschale Flüssigkeitssubstitution aus und plädiert für ein individuelles Vorgehen, da es keine ausreichende Evidenz für eine Verbesserung renaler Endpunkte gibt, jedoch Hinweise auf potenzielle kardiale Nebenwirkungen – Ziel ist die klinische Euvolämie. Sollte eine Flüssigkeitssubstitution gewünscht sein, müssen die Risikofaktoren für einen negativen Effekt einer Volumenexpansion geprüft und gegen den potenziellen Nutzen abgewogen werden, dies beinhaltet die Erfassung von insbesondere kardiovaskulären Komorbiditäten sowie eine Evaluierung des Volumenstatus. In diesem Fall empfiehlt die Arbeitsgruppe, eine orale Gabe vor einer intravenösen zu favorisieren.

Für ambulante Untersuchungen werden dazu folgende Screening-Fragen vorgeschlagen:

- Hatten Sie in den letzten Tagen Fieber oder Durchfall?
- Nahmen Sie in den letzten Tagen weniger als 1 l Flüssigkeit täglich zu sich?
- Haben Sie heute noch nichts getrunken?

Wird eine dieser Fragen mit „Ja“ beantwortet, kann eine Flüssigkeitszufuhr vor und nach der Untersuchung empfohlen werden.

### Dialyse

Ein prophylaktischer Einsatz einer NET (Hämodialyse oder Hämofiltration) zur Verhinderung eines CA-AKI sollte nicht erfolgen [61]. Eine systematische Überprüfung aus dem Jahr 2006 und eine größere Folgestudie fanden keinen Nutzen einer NET bei AKI nach KM-Gabe [62]. Die Arbeitsgruppe rät von einem „präventiven“ oder „therapeutischen“ Einsatz einer NET explizit ab.

### Mögliche Wechselwirkungen mit anderen Arzneimitteln

Aufgrund der seltenen Komplikation einer Metformin-assoziierten Laktatazidose (MALA) bei AKI wurde in früheren Guidelines zu einer Unterbrechung der Metformin-Einnahme vor einer ICM-Gabe geraten. Aktuelle Richtlinien empfehlen bei intravaskulärer ICM-Administration die Fortführung der Metformin-Einnahme, da das Risiko einer MALA weniger die eGFR als vielmehr ein niedriger systemischer arterieller Blutdruck oder eine Infektion ist [8]. Nephroprotektive Medikamente wie RAAS-Blocker und SGLT2-Inhibitoren sollen üblicherweise nicht vor KM-Gabe pausiert werden, auch eine stabile Diuretikatherapie muss periprozedural nicht unterbrochen werden [2]. Potenziell nephrotoxische Substanzen wie NSAR sollten nach Möglichkeit 24–48 h vor und 48 h nach KM-Gabe pausiert werden [63]. Aspirin ist zwar formal auch ein NSAR, wird aber in kardiovaskulären Indikationen wegen seiner plättchenhemmenden Funktion in einer niedrigen Dosis (üblicherweise 100 mg pro Tag) verordnet und braucht *nicht* pausiert werden.

## Kontrolle der Nierenwerte nach Kontrastmittelgabe

Eine SCr-Messung nach ICM-Administration wird nur bei einer eGFR ≤ 30 ml/min/1,73 m^2^ empfohlen. Das SCr steigt nach jeder stattgehabten Schädigung erst zeitversetzt, eine Kontrolle sollte laut Guidelines daher 48–72 h nach ICM-Administration erfolgen [2, 8]. Allerdings wirft diese starre Empfehlung praktische Fragen auf, v. a. wo diese Laborkontrolle erfolgen soll und welche Konsequenz sich ggf. aus einem moderaten Kreatininanstieg ergibt (die Einschränkungen dieses Biomarkers ohne Einbezug der Klinik wurden weiter oben diskutiert). Nach Meinung der Arbeitsgruppe kann eine Kontrolle der Nierenwerte bei RisikopatientInnen nach einigen Tagen nach klinischer Maßgabe erfolgen. Diese Empfehlung entspricht im Wesentlichen der empfohlenen Vorgehensweise im kanadischen Konsensus, in dem eine Laborkontrolle nur im Fall einer i.a.-KM-Gabe bei PatientInnen mit einer eGFR ≤ 30 ml/min/1,73 m^2^ empfohlen wird [2]. Bei den übrigen PatientInnen wird das CA-AKI-Risiko als so gering eingeschätzt, dass Routinetests nicht gerechtfertigt sind [2, 64].

## Gadoliniumhaltige Kontrastmittel (GBCA) bei CKD

### Assoziation mit nephrogener systemischer Fibrose (NSF)

GBCA werden seit 1988 in der MR-Bildgebung eingesetzt und haben zu einer deutlichen Verbesserung der Bildkontraste geführt. Im Jahr 2007 wurde erstmals ein Zusammenhang zwischen einer Dermatofibrose und der Administration von MR-Kontrastmitteln bei DialysepatientInnen beschrieben [65]. Nachfolgende Studien zeigten, dass es sich bei diesem GBCA-assoziierten Krankheitsbild um eine potenziell lebensbedrohliche systemische Fibrose handelt, bekannt als nephrogene systemische Fibrose (NSF) bei DialysepatientInnen bzw. PatientInnen mit fortgeschrittener CKD. Die Analyse der weltweit ca. 500 aufgetretenen NSF-Fälle hat gezeigt, dass das Auftreten dieser Entität in engem Zusammenhang mit der Molekülstruktur der applizierten GBCA steht. Da das ungebundene Gadoliniumion sehr toxisch wäre, muss es an Liganden gebunden werden. Abhängig von der Molekülstruktur werden lineare und makrozyklische GBCA unterschieden, wobei Letztere hinsichtlich einer sog. Transmetallierung sehr stabil sind, und das Gadoliniumion im Chelat nicht einfach gegen körpereigene Metallionen ausgetauscht werden kann. Dieser Mechanismus wird derzeit in der Pathophysiologie der NSF als ursächlich erachtet. Die Stabilität der GBCA hängt auch von der elektrischen Ladung der Moleküle ab, wobei ionische Moleküle stabiler als nichtionische sind. Da sämtliche NSF-Fälle nach Administration von linearen GBCA oder „confounded“, d. h. nach Mehrfachanwendung von linearen und makrozyklischen KM, aufgetreten sind, wurden die GBCA von der ACR und dem CMSC der ESUR in *Low*- und *High-Risk*-Substanzen eingeteilt (Tab. 4). Gadoxetsäure Gd-EOB-DTPA (Primovist®) ist ein leberspezifisches lineares GBCA mit größtenteils biliärer Ausscheidung und wurde 2024 mit dem Update der *ACR Guidelines* in die Gruppe der *Low-Risk*-GBCA eingestuft [10]. In Europa wurde die Zulassung der *High-Risk*-GBCA (Gruppe I) von der EMA (Europäische Arzneimittelagentur) 2017 zurückgezogen. Seitdem wurden keine neuen NSF-Fälle mehr berichtet.

**Tab. 4:** ACR-Klassifikation und Molekülstruktur von GBCA

ACR-Gruppe	Chemischer Name	Handelsname	Struktur	Molekülladung
Gruppe I*	Gadodiamide	Omniscan	Linear	Nichtionisch
	Gadopentetate dimeglumine	Magnevist	Linear	Ionisch
	Gadoversetamide	OptiMARK	Linear	Nichtionisch
Gruppe II	Gadobenate dimeglumine	MultiHance	Linear	Ionisch
	Gadobutrol	Gadovist	Zyklisch	Nichtionisch
	Gadoteridol	ProHance	Zyklisch	Nichtionisch
	Gadoterate meglumine	Dotarem	Zyklisch	Ionisch
	Gadopiclenol	Elucirem, Vueway	Zyklisch	Nichtionisch
	Gadoxetate disodium	Primovist	Linear	Ionisch
Gruppe III	Nil	Nil	NA	NA

Nebenbemerkung: Primovist wurde bis 2024 als Gruppe III GBCA geführt

* Gruppe I GBCA sind gemäß EMA seit 2017 in Europa nicht mehr zugelassen

In einem rezenten Update der ACR wird bei PatientInnen mit Nierenversagen nach GBCA-Gabe keine dringende oder außertourliche Dialysebehandlung empfohlen [10].

### GBCA und Nephrotoxizität

Obwohl ursprünglich GBCA als nicht nephrotoxisch erachtet wurden, haben präklinische und klinische Studien gezeigt, dass GBCA durch Osmotoxizität das Potenzial für ein AKI beinhalten [66–68]. Eine Metaanalyse von 59 Studien mit 1142 eingeschlossenen PatientInnen hat bei i.a.-Administration von GBCA eine CA-AKI von 5,95 % gezeigt [69]. Fallberichte einer GBCA assoziierten Nephrotoxizität sind immer verbunden mit der i.a.-Administration einer hohen Dosis von GBCA im Rahmen von angiographischen Anwendungen bei PatientInnen mit CKD [70]. Da es sich hier hauptsächlich um Fallberichte und retrospektive Studien mit fehlenden Kontrollgruppen handelt, ist es schwierig, einen definitiven kausalen Zusammenhang zu beurteilen [71–73]. Das CMSC der ESUR erachtet das CA-AKI Risiko als sehr gering, wenn GBCA in der zugelassenen Dosierung verwendet werden [8]. Low-Risk-GBCA (Gruppe II) können daher auch bei niedriger eGFR und bei DialysepatientInnen angewendet werden. Eine eGFR-Bestimmung vor Gabe von GBCA der Gruppe II ist nicht erforderlich.

## Zusammenfassung

Medizinischen Entscheidungen muss immer eine sorgfältige Nutzen-Risiko-Abwägung zugrunde gelegt werden. Bereits 2017 wurde in „Klug entscheiden“-Empfehlungen der Deutschen Gesellschaft für Innere Medizin bzw. Nephrologie postuliert, dass bei fortgeschrittener CKD der „*diagnostische Nutzen einer Röntgen‑/MRT-Kontrastmittel-Gabe mit potenzieller Reduktion der Morbidität und Mortalität gegenüber potenziellen Risiken stärker berücksichtigt werden sollte“ *[74]. Die aktuelle Datenlage hat einerseits das Bewusstsein für die negativen Konsequenzen einer hintangehaltenen KM-Gabe weiter geschärft, andererseits keine überzeugenden Belege für eine klare Kausalität von KM-Gabe und klinisch relevanten Folgeschäden geliefert. Es gilt daher, auch bei „Nierenkranken“ notwendige radiologische KM-Untersuchungen zeitgerecht durchzuführen und diese nicht aus übertriebener Furcht vor etwaigen renalen Konsequenzen zu verzögern oder zu unterlassen (für diesen therapeutischen Nihilismus bei Nierenkranken wurde von Chertow der Begriff *Renalism* geprägt) [75].

Die entsprechenden Konsensus-Statements der Arbeitsgruppe sind in Tab. 5 zusammengefasst.

**Tab. 5:** Konsensus-Statements bezüglich der Verwendung von KM bei CKD

Konsensus	Statement
Konsensus 1	Eine klinisch indizierte KM-basierte radiologische Untersuchung darf PatientInnen mit CKD nicht vorenthalten werden
Konsensus 2	PatientInnen mit einer eGFR ≤ 30 ml/min/1.73 m^2^ verbleiben als Risikopopulation für ein CA-AKI (insbesondere aufgrund der unzureichenden Datenlage)
Konsensus 3	Für diese Patientengruppe ist die Hydrierung die einzige potenziell zweckmäßige präventive Maßnahme. Ein individuelles Vorgehen ist notwendig, um eine Überwässerung zu vermeiden
Konsensus 4	Eine „Prophylaxe“ mit N‑Acetylcystein, Natriumbikarbonat oder anderen Substanzen wird nicht empfohlen
Konsensus 5	Im ambulanten Rahmen kann nach Ausschluss einer CKD (Screeningfrage) auf eine eGFR-Bestimmung verzichtet werden
Konsensus 6	Eine routinemäßige Pausierung einer kardiorenal protektiven Therapie vor KM-Administration wird nicht empfohlen
Konsensus 7	Eine Kontrolle der Nierenwerte kann bei RisikopatientInnen einige Tage nach ICM-Gabe nach klinischer Maßgabe erfolgen
Konsensus 8	Bei i.a.-KM-Applikation sollten nieder- oder isoosmolare ICM in der geringstmöglichen Dosierung verwendet werden, bei i.v.-Gabe können übliche KM-Mengen verabreicht werden
Konsensus 9	Eine KM-Administration kann auch bei DialysepatientInnen mit Restnierenfunktion/Restdiurese erfolgen, eine Änderung des Dialyseplans nach KM-Gabe ist nicht erforderlich
Konsensus 10	Kommt es nach KM-Gabe zu einem AKI, sollte auch nach möglichen anderen Ursachen gesucht werden
Konsensus 11	Die von der EMA zugelassenen GBCA können auch bei hochgradig eingeschränkter Nierenfunktion angewendet werden. Eine Bestimmung der Nierenwerte ist demnach vor Anwendung nicht erforderlich

## Supplementary Information

Für den Druck in A4 optimierte Version von Abbildung 1.

## Data Availability

Alle dieser Arbeit zugrunde liegenden Daten sind in diesem Artikel enthalten.

## Abkürzungen

ACEi: Angiotensin-converting enzyme inhibitor
ACR: American College of Radiology
AKI: Akute Nierenfunktionsschädigung
AKIN: Acute Kidney Injury Network
CA-AKI: Kontrastmittelassoziierte akute Nierenschädigung
CI-AKI: Contrast-induced acute kidney injury
CIN: Kontrastmittelinduzierte Nephropathie
CKD: Chronische Nierenkrankheit
CMSC: Kontrastmittelsicherheitskomitee
CT: Computertomographie
CTA: CT-Angiographie
eGFR: Estimated GFR
ESUR: European Society of Urogenital Radiology
GBCA: Gadoliniumhaltige Kontrastmittel
GFR: Glomeruläre Filtrationsrate
i.a.: Intraarteriell
ICM: Jodhaltige Kontrastmittel
i.v.: Intravenös
KDIGO: Kidney Disease Improving Global Outcome
KI: Künstliche Intelligenz
KM: Kontrastmittel
MALA: Metformin-assoziierte Laktatazidose
MRA: MR-Angiographie
MRT: Magnetresonanztomographie
NAC: N‑Acetylcystein
NET: Nierenersatztherapie
NSF: Nephrogene systemische Fibrose
PC-AKI: Postcontrast acute kidney injury
RAS: Renin-Angiotensin-System
RRT: Nierenersatztherapie
SCr: Serumkreatinin
