# Supplementary material for: Iodinated and gadolinium-based contrast agents and kidney disease: consensus statement of the Austrian Society for Radiology (OERG) and the Austrian Society of Nephrology (ÖGN)
Source: Wien Klin Wochenschr. 2026 Oct 27;138(Suppl 16):923–36. [Article in German] doi: 10.1007/s00508-026-02822-9 (PMC13748118; doi:10.1007/s00508-026-02822-9)
Supplement: Supplementary file 1 [file 508_2026_2822_MOESM1_ESM.pdf]

Algorithmus
ICM-Gabe und Nierenfunktion

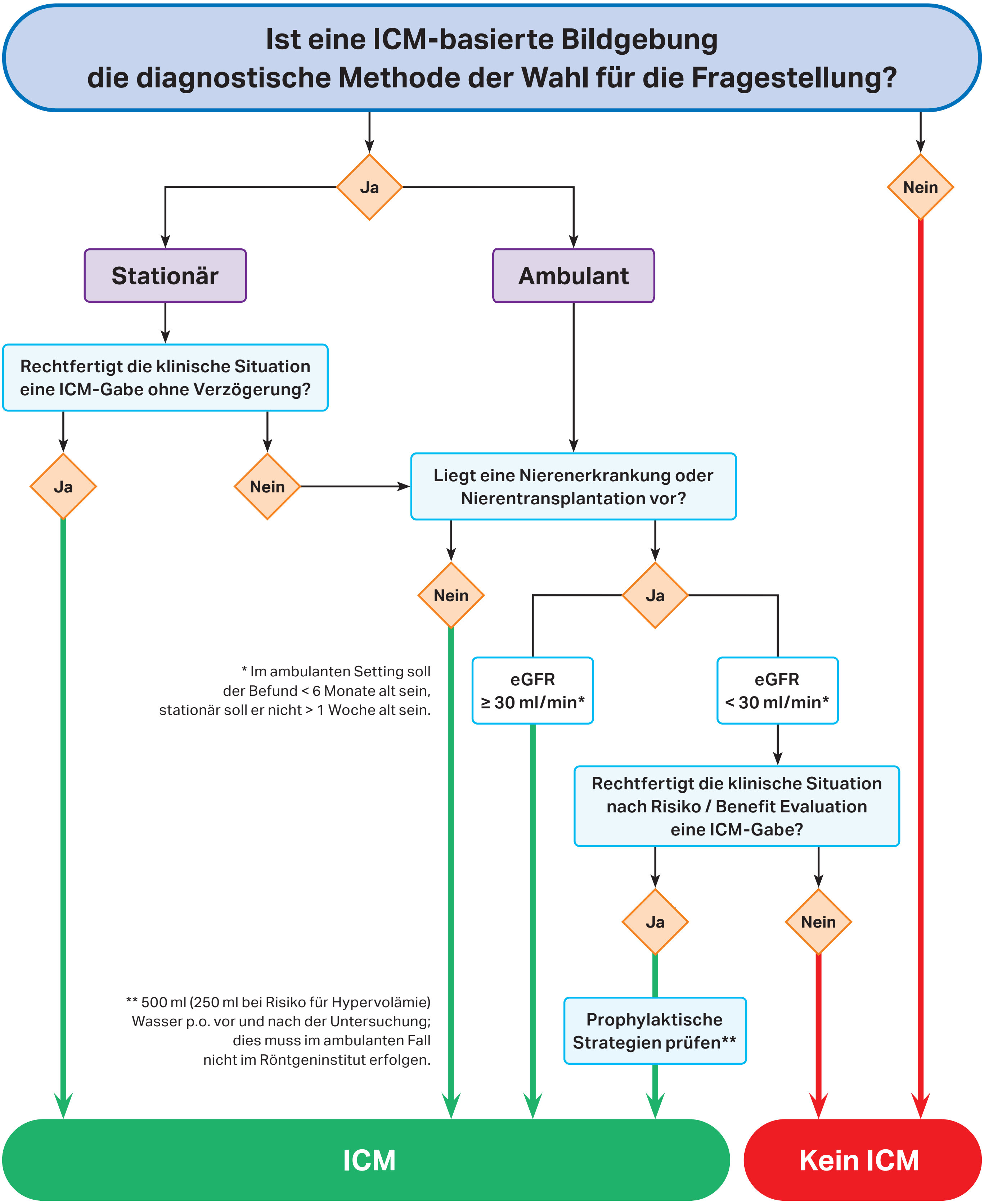

eGFR: estimated Glomerular Filtration Rate / geschätzte glomeruläre Filtrationsrate
ICM: Iodine Contrast Media / jodhaltiges Kontrastmittel

Gertraud Heinz, Martin Windpessl, Alexandra Ökrösi, Christian Krestan, Katharina Lampichler, Gregor Bond, Marcus Säemann, Katharina Hohenstein-Scheibenecker, Jodhaltige und gadoliniumhaltige Kontrastmittel und Nierenkrankheit: Konsensus-Statement der Österreichischen Gesellschaft für Radiologie (OERG) und der Österreichischen Gesellschaft für Nephrologie (ÖGN), Wiener Klinische Wochenschrift, 2026, DOI: 10.1007/s00508-026-02822-9
